# Herbal vaginal pessary induced acute renal failure

**DOI:** 10.4103/0971-4065.59338

**Published:** 2009-10

**Authors:** U. R. Onyemekeihia, C. O. Esume, C. O. Oladele, E. Oviasu

**Affiliations:** Department of Medicine and Pharmacology, University of Benin Teaching Hospital, Benin City, Nigeria; 1 Department of Medicine and Pharmacology, Delta State University, Abraka, Nigeria

**Keywords:** Alternative medicine, dilemma, herbal vaginal pessary, ignorance, renal failure

## Abstract

In Africa, the use of traditional herbal remedy is widespread. Acute renal failure (ARF) is one of the most serious complications. The use of herbal remedies (mostly orally) accounts for nearly 35% of all cases of acute renal failure in Africa. Development of renal failure following herbal vaginal pessary is rarely reported. In November 2003, a 35-year-old Nigerian female who is a petty trader and a primary school leaver with three children (all males) presented to us in the renal unit with oliguric ARF induced by herbal vaginal pessary. She had sought this alternative medicine in an attempt to have a female child as all her three children are males. Her condition was managed accordingly and required three sessions of hemodialysis. She started diuresing on the eighth day of admission. This case presentation highlights the potential tragedies of herbal preparation, of note, that herbal vaginal pessaries are as deleterious as the oral preparations, and that the dilemma of ignorance is still prevalent in our society.

## Introduction

Virtually, every culture and civilization throughout history has used a range of plants or plant derivatives for the prevention and treatment of diseases.[1] Recently, there has been an upsurge in preferential use of herbal medicines, many preparations of which can be found in the global pharmaceutical market.[2]

The popularity of traditional herbal medicines comes from the general belief that herbs are “natural” (which supposedly connotes “better”), more gentle and less toxic than synthetic western medicines[3] The most frequently reported conditions for which these herbal medicines are used include impotence, musculoskeletal problems including back and neck pains and body slimming.[4]

The use of traditional herbal medicines has no racial, geographical or social bounds. For example in 1999, 40% of Americans sought alternative medical treatment. This was more than the visits paid to conventional primary care physicians.[5]

Though many patients use alternative medicine, only 30% ever admit it to their health care professionals.[6] Also of concern is the fact that an estimated 15 million adults in USA alone are taking prescription drugs and herbal medicines concurrently, with the possibility of drug Interaction.[6]

In Africa, the use of traditional herbal remedies is widespread[1]. Numerous complications can arise from the use of these herbal preparations, ARF being one of the most serious complications. The use of herbal medicine (mostly orally) accounts for about 35% of all cases of ARF in Africa.[7] Development of ARF from use of herbal vaginal pessary is not common.

The aim of this communication is to highlight renal failure as a potential consequence of use of herbal preparations, and of note, that herbal vaginal pessaries are as deleterious as the oral preparations.

## Case Report

A 35-year-old Nigerian female who is a petty trader and a primary school teacher, presented to us in the renal unit of the hospital in November 2003 with a-ten day history of inability to pass urine, and an eight day history of vomiting and hiccupping.

The patient, who is not a known hypertensive or diabetic, had been in apparently good health until fifteen days before presentation when she inserted herbal vaginal pessary on the advice of a herbalist, in an attempt to have a female child, as all her children so far had been males. Five days after the herbal vaginal pessary insertion, she became anuric and two days after, she started vomiting and hiccupping. She had remained anuric until the eight day on admission when she progressively started making urine. There was no history of ingestion of oral herbal preparation, no diarrhea, facial or leg swelling nor hematuria. She had no symptoms of renal disease in the past. Her husband is a commercial taxi driver. She neither drinks alcohol nor smoke cigarette; and no family history of renal disease.

Examination revealed a young lady who was actually ill looking in mild respiratory distress. She was pale, anicteric, and afebrile to touch. There was no peripheral edema. Her pulse rate was 78 beats per minute, blood pressure was 120/70 mmHg. Her apex beat was not displaced. Fundoscopy was unremarkable. There were also no remarkable findings in the chest and abdomen. She was conscious and alert and there was no asterexis.

Laboratory investigation revealed 2+ proteinuria, many red blood cells (she was menstruating at that time) on urine microscopy, and culture yielded no growth after 48 h of incubation. Packed cell volume was 19%, total white cell count 7900/mm^3^.

Retroviral screening test, hepatitis B surface antigen and hepatitis C viral antigen were all negative.

Renal ultrasonographic scan revealed normal renal sizes of 11.5 × 4.5 cm and 12.0 × 5.6 cm for the right and left kidneys respectively. There was increased echogenicity with marked reduction of the corticormedullary differentiation. The serial serum electrolyte, urea and creatinine values are as indicated in Table 1.

**Table 1:** Serial serum electrolyte, urea and creatinine results

Date	Urea+ (Mg/dl)	Na‡ (Meq/l)	K§ (Meq/l)	HCO_3_ (Meq/l)	Cl± (Meq/l)	Creatinineς (Mg/dl)
Post dialysis						
16/11/03	183	138	5.2	18	109	9.8
20/11/03	82	138	3.2	20	108	7.1
Clinic appointment						
2/12/03	100	138	3.4	18	102	8.0
4/12/03	60	138	3.4	20	100	5.1
10/12/03	54	138	3.2	25	106	4.3
12/12/03	50	138	3.4	28	102	4.3
22/1/04	54	134	3.4	22	108	4.4
Last visit						
19/3/04	52	133	3.4	24	106	4.2

+ Normal range for urea is 10-50 mg/dl;

‡ Normal range for Na is 137-149 Meq/l;

§ Normal range for K is 3.8-5.1 Meq/l; Normal range for HCO3 is 24-34 Meq/l;

± Normal range for Cl is 97-10 Meq/l;

ς Normal range for creatinine is 0.6-1.1 mg/dl

Her creatinine clearance was 20 ml/min; and her packed cell volume at last visit remained at 27%.

She was treated with an average dose of intravenous frusemide of 100 mg every 12 h until she started diuresing. Strict input and output fluid chart was observed. She had transfusion of 2 units of packed cells (post transfusion PCV = 27%) and was subsequently placed on hematenics. She required three sessions of hemodialysis until she started diuresing, and blood urea and creatinine levels dropped to an acceptable level as shown in Table 1. She was discharged home after thirty-three days on admission. When she was last seen in outpatient clinic on the 19^th^ March 2004, she was clinically stable but her renal function test results, as shown in Table 1, still revealed elevated serum creatinine, and low creatinine clearance. Subsequently, she was lost to follow up. All attempts made to contact her using her residential address failed as she was said to have relocated elsewhere.

## Discussion

This case report describes a young lady of low socioeconomic and educational background, who had a sudden deterioration of her renal function following the insertion of herbal vaginal pessary, because of the desire to have a female child. But quite worrisome is the fact that after more than 12 weeks of initial acute renal failure, her serum creatinine remained elevated with a reduced creatinine clearance. What could be the possibilities? It could be that the acute renal failure precipitated by the herbal vaginal pessary was superimposed on a chronic renal impairment. However, the renal sizes remained normal, except for increased cortical echoes and the reduction of corticomedullary differentiation. The most likely possibility is that she had ARF following the use of the herbal vaginal pessary, which now has progressed to a chronic renal impairment. Most patients who survive an episode of ARF recover sufficient function to live normal lives. However, 50% have subclinical functional defects in glomerular filtration, tubular solute transport, H^+^ secretion and urinary concentration mechanism and glomerular or tubulo-interstitial scaring on renal biopsy.[8,9] Another possibility is the development of acute cortical necrosis. Patients with diffuse cortical necrosis do not recover renal function, whereas patients with patchy necrosis can lead to partial recovery.

Acute renal failure (ARF) is irreversible in approximately 5% of patients, usually as a consequence of complete cortical necrosis and requires long-term follow up with the possibility of long-term renal replacement therapy with dialysis or transplantation.[8,9]

An additional 5% of patients suffer progressive deterioration in renal function after an initial recovery phase, probably because of hyperfiltration and subsequent sclerosis of remnant glomeruli.[9]

This patient may just as well have fallen into any of these last two categories. Many herbal preparations may contain heavy metals (lead, mercury, arsenic) and other agents that are well known for their nephrotoxic potential.[10] Chen *et al.,* reviewed 58 cases of nephrotoxicity from Chinese herbs and three likely outcomes were revealed.[11]

1. Nephropathy with acute tubular necrosis and acute renal failure (7%).
2. Tubular dysfunctional nephropathy characterized by tubular degradation with atrophy and renal tubular acidosis and/or Fanconi syndrome (12%).
3. Chronic Nephropathy with renal interstitial fibrosis with sparse mononuclear cell infiltrates and chronically progressive renal failure (81%).

Other studies have suggested that tubulo-interstitial disease has a major impact on the progression of renal failure.[12] It was not possible to identify and analyze the component of the herb that was involved in this case because herbal prescriptions are treated as trade secrets in Nigeria. Also, the analysis of herbal medicine is difficult because of the complexity and variety of available formulations. Renal biopsy was not done in this case as the patient refused to give consent even after extensive counseling; therefore, the exact histopathological change in the kidney is unknown.

Several lessons should be learned from this patient. First, herbal vaginal pessaries are as deleterious as oral preparations and renal failure could be a complication. Second, the dilemma of ignorance is still prevalent in our society. The crave for particular sex of baby, which is common in our society, necessitated the use of this herbal remedy, with the consequent renal tragedy. There is need to put in place procedures for identification and quality control of herbal medicines stringently in developing societies like ours. We recommend that awareness campaign about the potential dangers of indiscriminate herbal medicine usage should be organized.
